# Adolescents’ and Parents’ Perspectives on Using the MedSMARxT Families Intervention in Emergency Departments for Opioid Medication Safety Education: Mixed Methods Study

**DOI:** 10.2196/68814

**Published:** 2025-06-26

**Authors:** Olufunmilola Abraham, Sara Nadi, Irene Hurst

**Affiliations:** 1 Pharmacy Practice Science, College of Pharmacy, University of Kentucky, 789 S. Limestone Street, TODD 292K, Lexington, KY, 40536, United States, 1 859-562-2766; 2 Social and Administrative Sciences Division, School of Pharmacy, University of Wisconsin–Madison, Madison, WI, United States; 3 Division of Pediatric Emergency Medicine, Department of Emergency Medicine, University of Wisconsin–Madison, Madison, WI, United States

**Keywords:** opioid medication safety, family medication safety, educational intervention, serious games, emergency department, MedSMA℞T, MedSMARxT

## Abstract

**Background:**

The opioid crisis has significantly impacted adolescents and their families. This is attributed in part to increased opioid prescriptions in pediatric emergency departments (EDs) due to acute pain conditions and injuries. Although EDs frequently prescribe opioids, no effective preventative interventions have been implemented to educate adolescents and their families on safe opioid use. This study evaluates the MedSMA℞T Families intervention, which consists of an engaging serious game, Adventures in PharmaCity, and a personalized Family Medication Safety Plan (FMSP) with the aim of reducing opioid misuse and promoting opioid medication safety. The MedSMA℞T Families intervention was developed to educate adolescents and adults prescribed opioids on safe practices such as opioid storage and disposal.

**Objective:**

This study aimed to explore and characterize adolescents’ and parents’ experiences and perspectives on implementing the MedSMA℞T Families intervention in the ED to improve opioid education and safety among adolescents.

**Methods:**

A total of 93 participants, including 16 children and 77 parents, were recruited from the pediatric ED at a tertiary academic hospital to play the MedSMA℞T game in the ED. A total of 16 participants, including 8 children and 8 parents, were followed up with interviews to gather qualitative feedback. Participants engaged with the MedSMA℞T game—Adventures in PharmaCity—and the FMSP. Data were collected through gameplay observation and 75-minute semistructured interviews via Zoom. Quantitative in-game data were analyzed using descriptive analysis and qualitative data were analyzed using thematic analysis with NVivo (version 14; Lumivero).

**Results:**

Parents spent an average of 22.16 (SD 4.97) minutes playing the game, while children spent an average of 21.99 (SD 8.06) minutes. Families appreciated the game’s design and noted usability challenges and suggested enhancements for clearer gameplay instructions. Participants reported increased knowledge of opioid safety, highlighted the importance of communication with health care providers, and a desire for a mobile app to assist with medication management. The FMSP was perceived as valuable for promoting awareness of safe practices and connected well to the knowledge gained from the game.

**Conclusions:**

The MedSMA℞T Families intervention was well received as a beneficial educational tool to educate adolescents and their families on safe opioid use. Additionally, it highlights a clear need for more accessible digital tools to increase opioid education. This feedback indicates a strong interest in improving educational resources to ensure safe opioid management within families.

## Introduction

Pediatric emergency departments (EDs) are considered the best practice models for the emergency care of children [1-5]. Patients frequently visit the ED for acute pain [6], including headaches, back pain, stomach discomfort, musculoskeletal injuries, postoperative pain, and pain associated with cancer. The use of opioids, conventional nonnarcotic medications (such as acetaminophen and nonsteroidal anti-inflammatory medicines), and nonpharmacological treatments including distraction techniques are among the most common treatments used by emergency physicians for pain management. Despite alternative pain control methods, opioids are commonly used in and prescribed from the ED for moderate to severe pain [6]. Concordantly, adolescent prescription opioid misuse is an increasing concern in the United States [7]. Nearly 60,000 opioid-related pediatric visits occurred throughout the US EDs from 2014 to 2017 [8]. In 2017, a public health emergency was declared due to the increased use and overdose incidences of opioids [9]. It is posited that an increased regulatory focus on pain management, aggressive pharmaceutical marketing, and the initial low concern for opioid addiction from prescription opioids have contributed to this crisis [10-15].

The use of prescription opioids increases the risk of opioid abuse in the future [16-21]. Persons with opioid use disorder commonly report that they were first exposed to opioids through a legitimate prescription, frequently from an ED [16-21]. As a result, the future risks and exposure to prescription opioids have significantly increased for individuals in their youth and adolescence. From 2019 to 2020, prescription opioid-involved death rates increased by 17% [22,23]. Adolescents who report having a valid prescription for a narcotic painkiller by the time they are in the 12th grade are more likely to abuse these prescription drugs later on [24,25]. Youth who report using opioids for nonmedical purposes before reaching adulthood are similarly at risk for abuse [26]. The rate of opioid misuse and opioid-related deaths among pediatric patients has increased along with the rise in opioid prescribing [17], in addition to increased hospitalizations attributable to opioid use [27].

Given the opioid crisis among adolescents in the United States and the increased risk of opioid misuse in this age group, it is crucial to address these issues effectively. Past interventions aimed at minimizing the risks of opioid misuse among adolescents have included universal prevention programs such as the Strengthening Families Program and the Life Skills Training Program, which focus on enhancing protective factors and reducing risk [19]. However, selective prevention strategies specifically tailored for at-risk adolescents and emerging adults remain limited, as recent studies have shown that most interventions fail to address the unique needs and contexts of this population [19]. Our two-part intervention, MedSMA℞T Families, aims to reduce the epidemic of opioid misuse by educating adolescents and their families who have been prescribed opioids from the ED. This intervention consists of an adolescent-tailored serious game entitled MedSMA℞T: Adventures in PharmaCity, as well as a family-focused tool called the Family Medication Safety Plan (FMSP), which is a personalized tool for family education [28]. Together, these resources help increase awareness about safe opioid use and provide educational information on safe opioid storage and disposal. There has been previous work regarding the MedSMA℞T Families intervention, such as quantitative and qualitative work investigating parental, adolescent, and staff perceptions regarding the game. However, the focus of this paper is the implementation of the MedSMA℞T game in the ED setting, specifically exploring the perspectives of the end users of this intervention [29]. This study explores parents’ and adolescents’ perspectives on the implementation of the MedSMA℞T Families intervention in the ED by examining the perceptions of adolescents and their families regarding the MedSMA℞T game and the FMSP.

## Methods

### Recruitment

The University of Wisconsin-Madison Institutional Review Board approved this study, and written consent was obtained from all participants. Families were recruited from a pediatric ED at an academic hospital in Wisconsin. Eligibility criteria included the ability to speak, read, and understand English, access to a computer, tablet, or mobile phone with videoconferencing ability, and having been prescribed an opioid medication from the ED. Data were collected through mobile gameplay, surveys for demographic information, interviews, and the creation of an FMSP with child-parent dyads. Recruitment flyers were displayed in high-volume areas throughout the department. Emergency Department Research Coordinators presented the study and distributed fliers at pre-shift huddles. If a child between the ages of 12 and 18 years in the ED was prescribed an opioid medication, the Emergency Department Research Coordinator team screened and approached families for participation. If families consented, the research team contacted participants by email at least 3 times to arrange a Zoom gameplay and interview session.

**Figure 1.**
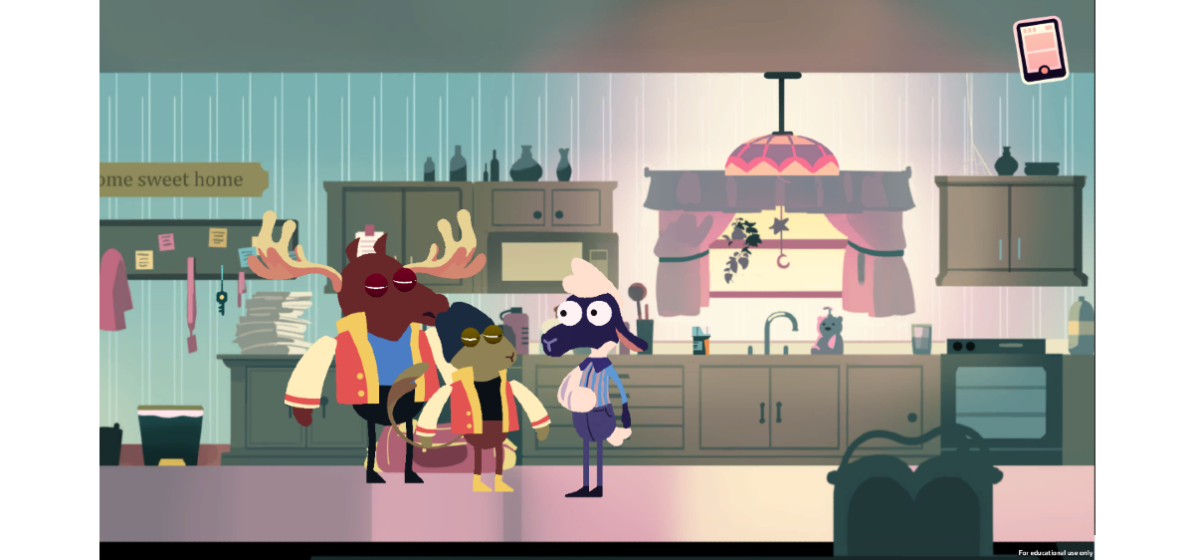
MedSMA℞T: Adventures in PharmaCity serious game.

The MedSMA℞T: Adventures in PharmaCity serious game (Figure 1) follows the story of an anthropomorphized sheep as they navigate making safe decisions regarding prescription opioids [28]. Correct opioid-related choices progress the story forward, while unsafe opioid-related choices rewind the story to allow the player to re-attempt the level and learn from their mistakes. In level 1, the player learns about safe opioid storage and the consequences of sharing opioids with friends. Level 2 provides background information and enhances the game storyline by adding a real-life scene about being in pain and forgetting there was an important assignment due that day. In level 3, the player learns not to take others’ prescription opioid medications. Level 4 teaches the player about the purpose of Narcan (a medicine that rapidly reverses an opioid overdose) and demonstrates the consequences of sharing opioids with others. Level 5 outlines safe opioid disposal. The game experience is inherently designed to improve adolescents’ decision-making about safe opioid use.

The personalized FMSP is a tool for families to record important information about their medications and create a plan for safe use, storage, and disposal [28]. The FMSP is designed to promote communication among families and providers or parent-child dyads as they complete and review the plan. It also serves as a tool for families to reference important medication information in one location and note any questions they have for their health care team.

Five MedSMA℞T Families features facilitate family communication and adolescent opioid safety. These include (1) engaging and interactive delivery through game-based learning; (2) realistic gameplay scenarios where adolescents and parents practice making safe medication choices in various community settings, including at school, in their neighborhoods, and at home; (3) a space for important medication information such as side effects, drug-drug interactions, dosage instructions, and reasons for use; (4) a section for proper storage and disposal information and planning; and (5) an area for positive communication, allowing families to ask questions about medications for their providers. The FMSP is displayed in Multimedia Appendix 1.

### Study Protocol

Data were collected between February 2023 and June 2023 until data saturation was reached. Similar to a previous study on pharmacist’s perspectives of MedSMA℞T, data saturation was achieved at 12 participants [30]. However, data collection continued to 16 participants to ensure no new data or themes emerged from the data set. Participants completed a survey about their demographic information before the gameplay. A member of the research team observed child-parent dyads as they played the game and became familiar with the FMSP for 30-minutes during the web-based meeting sessions. Following that, there was a 75-minute semistructured follow-up interview with the participants. The goal of observation during games was to record any problems that arose during gameplay and to step in if there was a technological error. Each session was documented by noting the occurrence of specific events, such as gameplay interruptions, participant questions, and instances of technological errors. Zoom was used to record both the interview and the gameplay. The interview audio recordings were professionally transcribed verbatim.

### Statistical Analysis

A descriptive analysis was conducted to analyze the quantitative in-game data. This included reporting the mean and the SD of the total time spent playing the game, the number of distinct levels played, and the number of distinct levels completed. It also included the number of opioid-related decisions or actions that resulted in game failure. Additionally, it reported the number of opioid-related decisions or actions that contributed to game progress and the total time spent playing levels 1‐5.

### Measures

A standard semantic inductive technique was used to examine qualitative data to investigate aspects associated with child-parent dyads’ viewpoints regarding the design and implementation of the intervention. With this method, implementation and iterative design patterns could be explored and defined based on participant answers.

One of the authors began by immersing in the interview transcripts and in-game data to gain a comprehensive understanding of the content. A coding framework was developed based on the initial read-throughs of the data. This included generating initial codes that encapsulated meaningful segments of the data using inductive thematic analyses via NVivo (version 14; Lumivero), a software program used for qualitative research. The initial codes were collated and organized into potential themes. This process involved examining the relationships between codes and identifying overarching patterns that captured the experiences of participants regarding the MedSMA℞T intervention. The themes were refined to ensure they accurately represented the data. This involved checking if the themes worked in relation to the coded extracts and the entire dataset. Each theme was clearly defined and named to reflect its content, ensuring clarity in communicating key findings. The final themes were reported along with illustrative quotes from participants to provide context and enhance the richness of the findings.

Reflexivity was considered throughout the study process. The authors engaged in regular discussions to reflect on their biases and preconceptions that could influence data interpretation. To enhance confirmability, the study team maintained detailed records of coding decisions and the evolution of themes. Dependability was addressed by ensuring that consistent procedures were followed throughout the study process through documentation of decisions made during data collection, analysis, and theme development, allowing for replication of the study if desired. While this study was conducted within a pediatric ED in Wisconsin, efforts were made to provide rich descriptions of the participants and the intervention. The diverse demographics of participants and the inclusion of both adolescents and parents enhance the transferability of our results.

### Ethical Considerations

This study has been reviewed and approved by the Institutional Review Board of the University of Wisconsin-Madison (2022‐0895). Informed consent was obtained from participants during the primary data collection process. Study data are anonymized and deidentified to protect the privacy and confidentiality of all human participants involved. Data handling processes are in place to ensure no identifiable information is disclosed. Participants in this study received compensation of US $40 gift card for their participation in the research.

## Results

### User Statistics

A total number of 93 participants, including 16 children and 77 parents, played the MedSMA℞T game in the ED and provided the in-game data. The mean age among children was 15 years, 68% (n=11) of children were females, 27% (n=4) of children were males, and 5% (n=1) of children were nonbinary. A total of 73% (n=12) of the children were White or Caucasian, 11% (n=2) of children were Black or African American, 9% (n=1) of children were Hispanic or Latinx, and 9% (n=1) of children were Asian. The mean age among parents was 46 years, 65% (n=50) of parents were females, 33% (n=25) of parents were males, and 2% (n=2) of parents were nonbinary. A total of 79% (n=61) of parents were White or Caucasian, 14% (n=11) of parents were Black or African American, and 7% (n=5) of parents were Hispanic or Latinx. Sixteen participants, including 8 children and 8 parents, were followed up with interviews. The average time spent on introducing the game and explaining the instructions on how to play was 15 minutes. The average time spent playing the game from in-game data for children was 21.99 (SD 8.06) minutes with an average of completing 3 out of the 5 levels in the game. On average, child participants made 3 opioid-related decisions/actions during the game process that led to game progress from correct decisions. Alternately, this group made 2 opioid-related decisions/actions that resulted in game failure indicating incorrect decisions, based on different scenarios in the game.

Regarding the in-game data for parents, the average time spent playing the game was 22.16 minutes (SD 4.97) with an average of completing 4 out of the total 5 levels in the game. On average, participants made 5 opioid-related decisions/actions during the game process that led to game progress from correct decisions. This same group made, on average, 3 opioid-related decisions/actions that resulted in game failure indicating incorrect decisions based on different scenarios in the game. Table 1 represents the in-game data analysis results for child participants and Table 2 represents the in-game data analysis results for parents.

**Table 1.** In-game data: children’s data (16 participants).

In-game data	Value, mean (SD)
Total time playing the game (minutes)	21.99 (8.06)
Number of distinct levels played	3.49 (1.54)
Number of distinct levels completed	2.97 (1.87)
Number of decisions/actions that lead to game failure	4.68 (2.82)
Number of opioid-related decisions/actions that lead to game failure	2.15 (1.42)
Number of decisions/actions that lead to game progress	3.57 (2.25)
Number of opioid-related decisions/actions that lead to game progress	3.48 (2.08)
Total time playing level 1 (minutes)	10.84 (6.58)
Total time playing level 2 (minutes)	1.26 (0.74)
Total time playing level 3 (minutes)	7.35 (5.65)
Total time playing level 4 (minutes)	1.28 (1.43)
Total time playing level 5 (minutes)	1.24 (1.92)

**Table 2.** In-game data: parents’ data (77 participants).

In-game data	Value, mean (SD)
Total time playing the game (minutes)	22.16 (4.97)
Number of distinct levels played	4.53 (0.85)
Number of distinct levels completed	4.16 (1.17)
Number of decisions/actions that lead to game failure	5.87 (2.52)
Number of opioid-related decisions/actions that lead to game failure	2.87 (1.45)
Number of decisions/actions that lead to game progress	5.00 (1.41)
Number of opioid-related decisions/actions that lead to game progress	5.18 (1.47)
Total time playing level 1 (minutes)	7.46 (2.90)
Total time playing level 2 (minutes)	1.40 (0.51)
Total time playing level 3 (minutes)	8.82 (2.51)
Total time playing level 4 (minutes)	1.77 (0.81)
Total time playing level 5 (minutes)	2.69 (2.77)

### Evaluation Outcomes

Four main themes were identified in the MedSMA℞T game. The first theme was titled “User Experience.” Three subthemes were then extracted from the data which were: “Usability,“ “Suggestions for Improvement,” and “Target Audience.”

Regarding “Usability,” parents mostly expressed having a hard time figuring out how to play the game and what they were supposed to do as the next steps. Children tended to have a more positive experience once they became familiar with the game instructions.

*I really liked the art style. It was really cute. I think the controls were easy to manage and to get the hang of.*

[Child 6]

The second subtheme was “Suggestions for Improvement” which included providing more interaction with scenarios throughout the game, more complexity in gameplay, more educational content on Narcan, and providing more guidance on emergency situations related to that.

The last subtheme was “Target Audience.” Feedback emphasized that the game’s target demographic seemed more suitable for children under 12 y old.

*…I think if you’re looking for giving it to demographics that are above the age of 12, or adults, it needs to be a game that would capture that attention.*

[Parent 5]

The second main theme identified was “Educational Learning and Value.” Two subthemes extracted from this theme were “Learnings” and “Perceived Goals.“ In the “learnings” category, participants highlighted significant takeaways from their experience playing the game and increasing their knowledge on important educational content. This included proper and safe disposal, storage methods, and side effects of opioids. One parent admitted to the following:

*I liked just learning more about the opioids. I did not know that you could take unused or expired prescriptions back to a pharmacy.*

[Parent 7]

Regarding the “Perceived Goals,” the educational aims were positively received such as teaching the significance of not letting peer pressure decide especially when children are at school, and educating on safe medication management.

*I thought it was a good way to teach people how to maintain and manage their medications and keep it out of the hands of other kids and the scenarios of not using other kids’ medications in school or in the home or on the bus.*

[Parent 4]

The third theme included “In-Game Elements” with subthemes of “Character Perception” and “Scenario Evaluation.” For “Character Perception,” while the child participants appreciated the realistic and fun character design, several parents found them too juvenile and more appropriate and appealing for 12 years or younger.

In the “Scenario Evaluation,” participants perceived the game scenarios to be realistic and relatable, and simulated real-world situations specifically different scenarios that could happen at schools. This reportedly made it a more engaging learning experience.

*I feel like they were pretty good and just showing different situations that you could easily have any common ones at school.*

[Child 8]

The last theme recognized was “Educational Gaming Experiences” with 2 subthemes as “Interest in Educational Games” and “Perceived Value.” Regarding the first subtheme, “Interest in Educational Games,” general enthusiasm was found for educational gaming among both parents and children. Participants mostly indicated that playing educational games is enjoyable if they find an element of fun and engagement in the gameplay.

*I don’t mind playing games that are educational as long as they are fun.*

[Child 1]

According to the last subtheme of “Perceived Value,” participants valued the game’s educational potential and the effective incorporation of educational content in an engaging format that captures attention and interest while increasing knowledge and awareness.

*I’ve played some other educational games, and I think there are just many different pathways you can go down with different consequences.*

[Child 8]

Table 3 presents further feedback on themes, subthemes, and additional illustrative quotes for the MedSMA℞T game.

**Table 3.** Themes and subthemes for the MedSMA℞T game: parents and children data.

Theme and subtheme	Illustrative quotes
User experience	
Usability	"I thought it was hard to maneuver. It was also hard to understand what I was supposed to do. Once I got going, I kind of understood, but some of the verbiage in it was kind of, left me wondering what I should do." [Parent 1] “Well, I thought it was really cute and simple once I figured it out.” [Child 2]
Suggestions for improvement	"I think the Narcan wasn’t touched on, but I don’t think, I mean, people don’t, most people aren’t carrying Narcan around in their pockets. So, I don’t know maybe talking a bit more about what to do if somebody does take medication they’re not supposed to, like calling 911 if they’re unconscious, or you know, calling urgent care or something if they took something but they’re still talking to you.” [Parent 4] “Maybe a little bit more detailed instructions. Maybe like less like dialogue and more like direction kind of.” [Child 1] "Maybe if there were more like, paths to go through? Like, I feel like I said there were, I think I just kind of breezed past most of them pretty quickly. Maybe I would like if there were more like, I don’t know, difficult options that were like, it was harder to find the correct answer. So, then there was like, more interaction with the cutscenes and stuff.” [Child 5]
Target audience	"I mean, probably younger kids that are more versed in games and just learning about, you know, the, the basics like maybe, you know, more like the preschool ages, you know, elementary, like the really young that need to learn more about the risks and, and could find those characters more appealing.” [Parent 8] "The characters I feel are kind of childish, if you’re looking for like teens or above age groups. If you’re looking to get, like maybe 12 and under, I think they’re fitting for that.” [Parent 7]
Educational learning and value	
Learnings	“I thought you could flush down the toilet, but, I guess I learned that is probably a no-no.” [Parent 3] "It’s just a useful tool like, kind of approaches different scenarios and allows kids to think through and adults to think through, what should I do in this scenario?” [Parent 6] "I feel like it would be useful for people to learn about like drug use. So, they didn’t know much about that. Or if they’re going through something like this. I think it’s a fun way to get people to learn about it. And yeah, I think I did learn quite a bit, because I felt like I didn’t really know anything to begin with so yeah.” [Child 4] "Well, before I wasn’t really sure about, like, any of the side effects like of opioids, or like any of the treatments or cures of that. I kind of knew that prescriptions weren’t supposed to be shared, but like I just guess I didn’t really know, exactly, like, why, but I guess, like, I guess the game taught me that, like, you know, it’s more because of like, different people need different amounts of stuff. And for one person, it can be an overdose can be like, way lower than with another person. And then also, I didn’t really know like, how strict you should be about disposing of the medications.” [Child 3]
Perceived goals	"Um, just how to not let peer pressure decide, you know, what’s right and wrong, and not to just assume that you should, just because something’s okay for you, that’s okay for everyone else, and to be responsible with things that are prescribed directly to you.” [Parent 5] "I feel like it could be a good educational experience for people who don’t have this awareness. I mean, whether it’s at the clinic or the pharmacy, when they go to pick up a prescription, it could be fun, especially if there’s kids in the house, or if it’s prescribed to a child or, or an adolescent.” [Parent 7] "I thought it was a good way to teach people how to maintain and manage their medications and keep it out of the hands of other kids and the scenarios of not using other kids’ medications in school or in the home or on the bus.” [Parent 4] “To probably to make like right choices and see what the choices are that you can make in situations.” [Child 7]
In-game elements	
Character perception	“I think the characters are kind of childish if you’re looking for a teen or above age groups.” [Parent 3] “I feel like they’re pretty realistic to an actual, like, high school student.” [Child 1] "I liked kind of how they weren’t like human. I think that made it a little bit more fun. I don’t know, I guess like, I did like the storyline where like, he had to do the speech and like, he was a bit nervous and stuff. And then, like, how he was in pain there. And then like, the dilemma of him, like, should I take this? Or should I not?” [Child 3]
Scenario evaluation	"I thought they were really good. Um, I guess the friend offering medicine and then the person asking for medicine were very salient points.” [Parent 8] “I liked the scenarios. I thought they were all really good. Um, yeah, I guess the characters were cute.” [Parent 6] "I felt like they were pretty like realistic to what could possibly happen in real life revolving around like a medication or drug.” [Child 3]
Educational gaming experiences	
Interest in educational games	"I don’t mind them if they are something that you know, is kind of that fast moving or keep your interest kind of thing. I don’t mind educational games. But, like I said it would have to kind of keep my interest.” [Parent 4] “I think they’re great.” [Parent 2] “I think they’re okay. They’re like fun and not boring.” [Child 5] "I think that it’s great if the game can be like, fun and educational at the same time. But a lot of the times when I’m playing games, I’m more in it for the fun rather than the education.” [Child 7]
Perceived value	"I mean, I would think word games to be educational. But I like it. I’d like I mean, I know that like playing word games keeps your mind sharp, especially as you get older.” [Parent 1] "I like how um it teaches you like stuff in a different way. And um I sometimes don’t like the topic that we learn about but.” [Child 6]

Three main themes were identified as a result of thematic analysis for the FMSP. The first theme was “Routine Medication Management” with 2 subthemes, “Medication Safety Practices at Home” and “Communications with Healthcare Providers.” “Medication Safety Practices at Home” reported various practices in terms of the storage and disposal of opioids to maintain safety and secure medication management strategies.

*I have a lockbox that has some actually, old medicines that need to be taken to a drop box in it. But otherwise, everything is pretty accessible.*

[Parent 5]

*We keep them in a cabinet or a special door. We don’t really take them unless we need to.*

[Child 7]

For “Communications with Healthcare Providers,” parents highlighted the significance of effective communication with health care providers regarding medications and different questions they may have. They mostly reported using digital platforms such as MyChart to communicate with their doctors and reaching out to their pharmacists asking prescription-related questions.

*…it’s mostly by MyChart, you can just, send the doctor a message and they’re pretty fast at responding back. Unless it’s something you need an immediate [response], then you call.*

[Parent 4]

*I used to talk to the pharmacist about it. I usually also ask about if I am on any other medications or vitamins if it is all okay, whether or not I have to take it with food, trying to figure out a schedule of when I need to take it, writing it down as to when I took it last, if I forget, I can look back at that log to see when the last time is that I took it.*

[Parent 2]

The second theme was “FMSP Usability,” including a subtheme identified as “FMSP Implementation Setting.” Participants had a perception that health care providers would play an important role in implementing the FMSP. Many participants shared different ways for providing information about the FMSP to patients, such as adding prescriptions from doctors’ offices, implementing through MyChart in after-visit summaries, as a printout in your medication bags at the pharmacy, or being advertised at schools.

*… it could be something in your after-visit summary that they print out or put on MyChart for you. Your pharmacist could print one out and put it in your medication bags.*

[Parent 6]

*I feel like pharmacies or doctor’s offices [would] be good? Even if there was an ad or commercial, could bring it up at school too, or health class or advisory?*

[Child 6]

The second subtheme was “Recommendations for Platform Enhancement.” Participants provided insightful comments on improving the usability of the FMSP. Many stated that an app on mobile devices would facilitate medication tracking, making it more convenient to have medication reminders and logs accessible.

*I think an app would be most useful because you have your phone with you all the time, and if you are away from home, and you have medication with you, that you take, you would be able to track it, when you took it. If you had any side effects, you could maybe even set an alarm on your phone, through the app that would tell you or remind you now is time to take your medication. and then you can maybe check it off so that you know that you took it at that time, and you can look back and see the times that you have taken it.*

[Parent 2]

A child also suggested improvements as follows:

*Well if it’s in an app, on a phone or computer, or if you have an Apple Watch.*

[Child 4]

The third and last theme identified was “FMSP Usage,” with 2 subthemes of “Awareness and Education“ and “The Correlation Between the MedSMA℞T Game and the FMSP.” For “Awareness and Education,” participants perceived the FMSP as a useful and beneficial tool for increasing their awareness and knowledge about medication safety practices such as safe disposal, storage, and use of opioids. Children also echoed the desire for similar tools for safety.

*Knowing where else to dispose of them. It’d be easier for me to go to the pharmacy because I go there to pick up medications and I can just dispose of them through the pharmacy. And for some people working through it with their pharmacist or even a pharmacy tech for there’s a lot of low health literacy out there.*

[Parent 4]

‘The Correlation Between the MedSMA℞T Game and the FMSP” subtheme indicated a perceived connection between the knowledge gained form the MedSMA℞T game and the principles of the FMSP.

*…it made it easier because already have to think about medication storage and disposal because you’d already gain some knowledge.*

[Parent 8]

Participants emphasized the educational value of the game and that learning from the game reinforced key safety messages, such as not sharing opioids and adhering to prescribed dosages. Table 4 presents themes, subthemes, and additional illustrative quotes for the FMSP.

**Table 4.** Themes and subthemes for the FMSP: parents and children data.

Theme and subtheme	Illustrative quotes
Routine medication management	
Medication safety practices at home	"So, the kind of non-prescription ones are in the cabinet. Um, the prescription ones are in a, in a cabinet they’re tucked away for just us and then when we are done using them or they need to be disposed of, I just kind of collect them in the safe until I know I’m gonna go somewhere to get rid of them so.” [Parent 1] “Normally know where it’s kept and when to take it.” [Child 3]
Communications with health care providers	"Usually through MyChart is kind of, you know, the way I do it, if it’s like more of an urgent matter then I would try calling them first if it’s like Um, I don’t know, I guess I haven’t really had a circumstance or then real urgent. I would call like a nurse on call or something. But I, otherwise it’s just been, like waiting for someone to revive you through like later.” [Parent 6] "Sometimes if it’s like, just a medication question like, Um, whether or not they have to have it with food or, or things that sometimes I’ll just call the pharmacy, and I’ll talk with them about that versus the doctor. You know, if it’s not clear on the paperwork that came home, the prescription or things, there’s a discrepancy of what we thought the doctor said, versus the paperwork says, I’ll usually reach out to the pharmacist.” [Parent 1] “I would ask about side effects, safety, dosage, time to take, storage.” [Parent 3] “Um probably just about like, how often I should be taking it and the side effects**.**” [Child 7]
FMSP^a^ usability	
FMSP implementation setting	"I would say both the doctor and the pharmacy. Of course, you would then have it actually, have it on your phone or whatever. But like, information about it would be useful both at the doctor and the pharmacy.” [Parent 1] "I mean, I think especially if it’s a high-risk medication, giving it to somebody when they pick up the medication from the pharmacy would be a good idea. And, and then I think maybe it’s more for children and their medications and management.” [Parent 8] "Um, I would say, you know, probably more like the, like pediatric, like the checkups, like when doctors’ offices, like when you would go in, when your kids are young like maybe first starting out like when, you know, medications like you might start getting some of those drugs prescribed.” [Parent 3] “At the pharmacy and doctor’s office.” [Child 2]
Recommendations for platform enhancement	"I think it depends on the person. I can see some people needing to have paper pencil, but then other people would just lose their papers. So, having it electronically, and everyone has a computer in their pocket all the time now. So, something that is mobile friendly or maybe even you could, you could develop an app.” [Parent 5] "So, I keep coming at this as helping other people, but you know, maybe having something on my phone, that would just make it right there. It could be one of those, you know, how families have their family sharing**.** It can be like a shared calendar, but a shared medication management app or something. And then the app could also have alarms to remind people to take their medications.” [Parent 2] “On a website? Maybe like an app or something?” [Child 3] “I think an app would be good.” [Child 1]
FMSP usage	
Awareness and education	"Yeah, I, I mean, I guess I, I was one of those that that you could flush it down the toilet. So, I mean, I guess, you know, just kind of knowing a little bit more like, what is safer for getting rid of.” [Parent 2] "Just to like keep it, like to use it. Make sure you’re using it safely and how to keep yourself and others safe.” [Child 4]
The correlation between the MedSMA℞T game and the FMSP	"Um, I guess I would just maybe be a little more, not that I blocked it away, Um, but just, you know, maybe, I guess maybe a little more verbalizing, you know, like talking to the kids more about usage, and, and just safety.” [Parent 5] "Um, I mean, just the kind of some of the, I mean, like I already knew, but it’s still reminder the common sense**,** like don’t share it. Um, you know, just don’t take, you know, more than prescribed because, you know, sometimes yeah, when you’re in a lot of pain, you sometimes you don’t, you don’t care because it hurt, you know, so that you, you might, I guess just do it without thinking, so I think just, you know, staffing and realizing to think a little bit more about things.” [Parent 3] "I think it looks good. I think it considers like all the different things you would need to know, to just kind of have it laid out.” [Child 2]

a FMSP: Family Medication Safety Plan.

## Discussion

### Principal Results

As the opioid crisis continues, opioid education and prescribing practices continue to be a highlight of investigation and improvement in EDs nationwide. This study sought to understand adolescents’ and parents’ perceptions of the MedSMA℞T Families intervention for both gameplay and the FMSP. This study addresses this gap by exploring their valuable and novel insights regarding this intervention. It also demonstrates the general acceptability and usefulness of the MedSMA℞T Families intervention in educating parents and children presenting to an ED about opioid safety. Considering the observations during gameplay, it is noteworthy that both parents and adolescents made errors, which provided significant opportunities for teaching and reinforcing important concepts related to opioid safety. These errors, rather than being viewed negatively, revealed crucial insights into the participants’ knowledge gaps and misconceptions regarding safe opioid use. The findings from our study suggest that users found the game straightforward and smooth to play once they became familiar with the instructions. Participants were willing to have direct communication with health care providers and use digital tools to ensure appropriate medication management and opioid safety. Respondents’ feedback suggested enhancements for the platform to be more user-friendly, flexible, and tech-savvy to support and facilitate medication management. Our results also indicate a strong desire for ongoing education regarding medications and especially opioid management.

### Comparison With Prior Work

A previous study was conducted to explore ED staff perceptions on implementing the MedSMA℞T Families intervention in the ED setting and assessing its feasibility within the context of the ED environment. It was reported that the game is more fun and age-appropriate for adolescents compared with the current educational materials used in the ED. ED staff believed that an interactive design and real-life scenarios helped with creating a unique approach to involve adolescents, as they are interested in technology-based games. Game availability as a phone app and translation to other commonly used languages among non-English speaking patients such as Spanish and Hmong were suggested. Additionally, it was reported that the FMSP is a good resource for families at home as it provides detailed information regarding safe opioid use, disposal, and storage that is often missed in patient education [31].

Results are also consistent with a previous study on assessing adolescents’ experiences and their suggestions for the use of the MedSMA℞T game. This identified that adolescents were successful in identifying the game’s aim which is promoting opioid medication safety. They had positive impressions of the game’s level, graphics, and characters. Participants recommended more instructions for how to play the game and create more game levels, which were implemented at that time [32].

In another study that explored parents’ perceptions of the MedSMA℞T game, participants expressed positive reactions to the game characters and scenes depicted in the game. They thought scenarios were appropriate and realistic for adolescents and raised their awareness about safe strategies to store and dispose of opioids. Similar to the current results in our study, parents of adolescents who were prescribed opioids in ED reported some challenges in navigating through the game. The slow pace of the game was viewed as a significant difficulty for gameplay. However, parents still recommended implementing the game in both health care and nonhealth care settings [33].

Pharmacists’ perceptions about the MedSMA℞T game have also been previously examined. Results highlighted the age-appropriate language of the game for adolescents and the use of relatable characters in the game design. Pharmacists valued the interactive nature of the gameplay, which led to active learning and recalling educational content [30].

Adolescents have also found the FMSP to be an acceptable and useful tool for opening conversations about opioids and other medications with their parents [34]. Parents’ also viewed using the FMSP to promote proper opioid prescription practices with adolescents as demonstrated in this study, and reinforced the significance of a personalized family plan for safe medication management and practices at home [35]. Similarly, pharmacists’ perceived the FSMP as a tool to encourage interactive opioid conversations between adolescents, families, and pharmacists as well. They believed that patients might use the FMSP as a visual cue to help think of what questions families should ask pharmacists [36].

Our study reinforces previous results that demonstrate significant gaps in knowledge regarding opioid safety among adolescents and their families. However, novel themes were also identified that emerged specifically from the ED setting, which differs from other settings in prior studies. While there are similarities between other previous studies and our study, findings suggest significant positive impressions and key recommendations for future improvement of the intervention. Our study also suggests that parents and adolescents found the interactive nature of the game more engaging and suitable for their learning needs compared with traditional educational materials. This aligns with findings from ED staff that indicated a preference for interactive, technology-based educational tools [31]. Participants also highlighted important considerations for leveraging health care professionals to implement the intervention in their health care settings. This would facilitate effective communication with health care providers and the use of this intervention for families.

Serious games are used as the main educational tool in the MedSMA℞T Families intervention as well as the research study by Aneni et al [37] and Pendergrass et al [38]. Adolescents, a demographic population, who do not typically use traditional instructional materials for medicines, were the target audience for all 3 interventions discussed in these studies. The gameplay’s interactive features encourage engagement and improve learning outcomes. In line with the goals of the MedSMA℞T game, the 2 other serious games also aimed to raise awareness about the risks of opioid misuse and safe storage and disposal. The 2 other studies used additional resources to strengthen the gaming experience, as the MedSMA℞T Families intervention incorporates the FMSP. These additional materials frequently support continuing education on safe medication practices and enhance family conversations.

Aneni et al [37] focus on older adolescents, possibly excluding the parental perspective [37], whereas the MedSMA℞T study specifically includes both adolescents and their parents, establishing a family-centered approach. The school-based health settings described by Pendergrass et al [38], on the other hand, place more emphasis on educational outcomes in school settings rather than on direct family interactions [38]. The MedSMA℞T game scenes and features encourage adolescents to learn by making mistakes and were specifically made to simulate real-life situations with the use of opioids. Aneni et al [37], on the other hand, investigates alternative design approaches or gaming mechanisms that prioritize role-playing or narrative components, altering how players engage with the content. Opioid medications are administered in a variety of contexts in these studies. Pediatric EDs, an acute care setting that might affect how information is received and the urgency attached to it, were used to test the MedSMA℞T Families intervention. Conversely, Aneni et al [37] and Pendergrass et al [38] explore settings such as schools, which can change the approach and interaction style influenced by the institutional environment and common teenage experiences.

### Limitations

As child-parent dyad interviews were recorded via Zoom, participants might have been more likely to respond in a socially desirable manner, as opposed to nonvisual phone interviewing. Another limitation is that all the participants interviewed were from one academic ED, which may not represent the sentiments of all EDs across the United States. Given the unique characteristics of ED settings and their vital role in intervention implementation, further research using a more diverse sample of ED settings is warranted to gain organizational-related information and adaptations. Additionally, one of the limitations is to address the feedback that the game was appropriate for kids younger than 12 years which could be improved in future versions of the game.

### Conclusions

In conclusion, participants found the MedSMA℞T intervention to be a useful educational tool for adolescents and families who were prescribed opioids from an ED about safe opioid use, storage, and disposal. By focusing on ED participants, we emphasize the unique context of the ED setting, which affects the attitudes and experiences of adolescents and their families. This study uniquely contributes to the literature by demonstrating how engaging and interactive educational tools, such as the MedSMA℞T game and the FMSP, can effectively support opioid safety education in a high-stress environment.

Participants offered insightful feedback on user experience and suggested future improvements regarding content and gameplay instructions. Participants appreciated the FMSP as an instructional tool. Future improvements were recommended, including making it available on mobile devices. There is a clear need for improved digital tools that promote opioid safety and highlight the significance of knowledge, instruction, and useful tactics for efficiently managing opioids and other medications in the home.

## Supplementary material

10.2196/68814 Multimedia Appendix 1 Family Medication Safety Plan.

10.2196/68814 Multimedia Appendix 2 Parent interview questions.

10.2196/68814 Multimedia Appendix 3 Child interview questions.

## Abbreviations

ED: emergency department
FMSP: Family Medication Safety Plan
