# Supplementary material for: Adolescents’ and Parents’ Perspectives on Using the MedSMARxT Families Intervention in Emergency Departments for Opioid Medication Safety Education: Mixed Methods Study
Source: JMIR Serious Games. 2025 Jun 26;13:e68814. doi: 10.2196/68814 (PMC12226961; doi:10.2196/68814)
Supplement: Multimedia Appendix 1 [file games-v13-e68814-s001.pdf]

Family Medication Safety Plan

The tool below will help you and your children think about medication use in and outside the home and create goals and rules that align with your family's values. Medications should work for YOU and work within YOUR family values and parenting style. When used thoughtfully and appropriately, medications can enhance quality of life and improve health outcomes. But when used inappropriately or without thought, medications may negatively impact your health and result in unintended harm. By creating a Personalized Family Medication Safety Plan, you can be more aware of using medications safety and responsibility to achieve positive health outcomes. To make YOUR family's Medication Safety Plan, start by entering your family's medication questions and information. This information will remain private and confidential.

Medication and Family Information:	***Dosage and Instructions***						
Family member name and age:	Reason for use	Dosage & How to take	Over-the-counter or prescription medicine?	Benefits	Potential side effects	Drug-drug interactions 2	Food-drug interactions 3
Medication 1 (Name, Brand, Generic)1:							
Medication 2 (Name, Brand, Generic):							
Medication 3 (Name, Brand, Generic):							
Medication Schedule							
**Scheduled time**	Monday	Tuesday	Wednesday	Thursday	Friday	Saturday	Sunday
Medication 1 Time:							
Medication 2 Time:							
Medication 3 Time:							
Proper Storage and Disposal	***Discuss plans for storage and disposal in your home and write in below***						
Where you store your medication							
Instructions for storage of your medication							
Expiration & time for disposal							
Proper disposal techniques 4							

CROME LAB
Collaborative Research On MEdication use & family health

Positive Communication	*Discuss positive communication for medication concerns and questions and fill in below*
Questions about medication	
Who and how to contact for questions about medications<br>Name:__________________Phone:___________	
Plan for medication use in school	
What to do in case of accidental use or overdose	
Other medication related concerns & questions	

1 For example, Advil is the Brand Name of the medication and Ibuprofen is the Generic Name
2 Drug-drug interactions mean how one medicine may react with another when taken at the same time
3 Drug-food/beverage interactions result from drugs reacting with foods or beverages. For example, mixing alcohol with some drugs may cause you to feel tired or slow your reactions. Other drugs may need to be taken with food.
4 The best way to dispose of your expired, unwanted, or unused medicines is at a drug take back site or your local pharmacy. You should not put any medication down the drain.

CROME LAB Collaborative Research On MEdication use & family health
