# Supplementary material for: Adolescents’ and Parents’ Perspectives on Using the MedSMARxT Families Intervention in Emergency Departments for Opioid Medication Safety Education: Mixed Methods Study
Source: JMIR Serious Games. 2025 Jun 26;13:e68814. doi: 10.2196/68814 (PMC12226961; doi:10.2196/68814)
Supplement: Multimedia Appendix 2 [file games-v13-e68814-s002.pdf]

Parent Interview Questions:
Part 1
Interview Discussion Guide- (30 minutes)

The first questions are about the game and elements of the game.

What did you think of the video game?

What did you like or dislike about the game, and why?

What changes could be made to improve the game?

Tell me how you feel about the characters in the game. (Prompt, if needed: Were they realistic? What did you like/dislike about them? How could we improve them?)

How did you feel about the scenarios presented in the game? (Prompt, if needed: Were they realistic? Engaging?)

Would you recommend this game to others, why/why not?

The following questions are about what you learned from the game.

What do you feel was the main goal of the game? How could the game be improved in order to meet this goal?

What did you learn from this game, if anything?

Now, I'd like to ask you some questions about your experience with games.

Do you have any experience with playing video games? Please tell me about your experience with video games.

(If they have little to no experience): Is there a reason you don't play video games? If yes, why not?

(If they have little to no experience): Is there anything that could be changed that would make you want to play them?

(If they do play games): What type of video games do you prefer?

What games are you currently playing? Why do you enjoy playing these games?

Do you play games on your phone / board games? If so, what games? Why do you play them?

How do you feel about playing games that are educational?

Have you played any other educational games? If so, what do you like or dislike about these games?

Is there anything else you'd like to add?

Part 2
Interview Discussion Guide- (1 hour)

First, we would like to ask you about medication safety.

What does “medication safety” mean to you?

Now, we would like to ask you some questions about the game.

Tell me about your experience playing the game.

What stood out the most when playing the game?

What thoughts or questions did you have about opioid use after playing the game? *(prompt: level 2, 3, 4.)*
Opioids are medications that are often prescribed by nurses or doctors to treat pain. Legal opioids are those that have been prescribed by a nurse or doctor.
FOR REFERENCE: Codeine (Tylenol #3), Fentanyl, Hydrocodone (Vicodin, Lortab, Lorcet, Norco), Hydromorphone (Dilaudid), Meperidine (Demerol), Methadone, Morphine (MS Contin), Oxycodone (OxyContin, Percocet), Tramadol (Ultram)

What thoughts or questions did you have about medication safety after playing the game? *(prompt: level 1 & 5)*

What else would you want to see in the game? (*Prompt: Is there anything you would change about this game)*

What would you want to get rid of in the game? What was not useful?

Next, we would like to ask you some questions about medication management practices in your home. (*Interviewer Note: If these questions don’t apply, ask them how they would go about medication management IF someone in the home was prescribed medications.)*

If you are prescribed a medication, how are you involved in managing this medication. *(Prompt, if needed: For example, are you the one who stores, gives out and/or reminds you to take the medication, etc.)*

Who in your home manages prescription medications? Over the counter medications? (*Note: clarify what over the counter medications mean. “Over the counter medications, or OTCs, are medications that you can buy off the shelf without a prescription.”)*

Who in your home has access to prescription medications? Over the counter medications?

What practices do you follow to safely store and manage medications in your home? *(Prescription, over the counter)*

Describe how prescription and over the counter medications are managed at school. What communication do you have with school staff? *(If no current medications, ask how they would do this)*

Who in your family is involved in speaking with your healthcare team (*doctors, nurses, pharmacists*) about medications? Describe how you and your family communicate with the healthcare team.

What questions do you think you would ask your healthcare team about your medications*? (Prompt: side- effects, proper disposal, drug-drug interactions, food-drug interactions etc.)*

Show the interviewees the FMSP template.

Thank you for completing the Family Medication Safety Plan. Please have the FMSP in front of you for reference for the following questions.
Tell us about your experience of completing the FMSP.

What challenges did you encounter while completing the FMSP? *(Prompt: was there a moment where you got stuck?)*

What aspects of the FMSP were not clear?

On a scale from 1-10 (*1 being **not at all** and 10 being **everyday***) how likely are you and your family to use the FMSP? And how would you use it?

How often would you update the FMSP?

What format would you like to see this guide in? *(paper-pencil, electronically)*

Where would you like it to be available? *(prompt if needed: pharmacy, doctor's office, etc)*

How would you like it to be available online? *(view, complete, print, update)*

What barriers might you experience to using the FMSP?

What might facilitate your use of the FMSP or make filling out the FMSP easier?

How did playing the MedSMA℞T game inform how you use the FMSP?

Do you have plans to change your medication practices in your home after playing MedSMA℞T?
If yes: What changes do you plan on making?

Is there anything else you would change? Anything else that is unclear? Is there anything you would add or remove?

Now we would like to ask you about filling out each section of the FMSP.

Tell me what you think about the Introduction Paragraph.

- Would you change any part of this? Is it clear? What questions do you have about this section?

Tell me what you think about the Medication and Family Information section.

- Would you change any part of this? Is it clear? What questions do you have about this section?

Tell me what you think about the Dosage and Instructions section.

- Would you change any part of this? Is it clear? What questions do you have about this section?

Tell me what you think about the Medication Schedule section.

- Would you change any part of this? Is it clear? What questions do you have about this section?

Tell me what you think about the Proper Storage and Disposal section.

- Would you change any part of this? Is it clear? What questions do you have about this section?

Tell me what you think about the “Discuss plans for proper storage and disposal….” section.

- Would you change any part of this? Is it clear? What questions do you have about this section?

Tell me what you think about the Positive Communication section.

- Would you change any part of this? Is it clear? What questions do you have about this section?

Tell me what you think about the “Discuss positive communication for medication concerns...” Section.

- Would you change any part of this? Is it clear? What questions do you have about this section?

Is there anything else you’d like to add?

Now we will ask about the opioid medication that was prescribed for (you OR your child), and how the MedSMA℞T intervention influenced your how (you OR your child) used it.

Were you or your child prescribed an opioid medication at discharge to take home?

(IF NO OPIOID PRESCRIPTION, ASK THE FOLLOWING QUESTIONS)

How has the MedSMA℞T game influenced the way you might use prescription opioid medications?

How has the FMSP influenced the way you might use prescription opioid medications?

How has the MedSMA℞T game influenced the way you might store prescription opioid medications?

How has the FMSP influenced the way you might store prescription opioid medications?

How has the MedSMA℞T game influenced the way you might dispose of prescription opioid medications?

How has the FMSP influenced the way you might dispose of prescription opioid medications?

Is there anything else you would like to add?

(IF YES, THEY WERE PRESCRIBED AN OPIOID, ASK THE FOLLOWING QUESTIONS)

Did you pick up the prescription opioid medication from the pharmacy? Why or why not? What was your thought process here?

How has the MedSMA℞T game influenced the decision to pick up the prescription opioid medication?

How has the FMSP influenced the decision to pick up the prescription opioid medication?

(ASK THE FOLLOWING QUESTIONS IF THE OPIOID PRESCRIPTION WAS PICKED UP)

How has the MedSMA℞T game influenced the way (you are OR your child is) using prescription opioid medications?

How has the FMSP influenced the way (you are OR your child is) using prescription opioid medications?

How has the MedSMA℞T game influenced the way you store prescription opioid medications?

How has the FMSP influenced the way you store prescription opioid medications?

How has the MedSMA℞T game influenced the way you dispose of prescription opioid medications?

How has the FMSP influenced the way you dispose of prescription opioid medications?

Is there anything else you would like to add?

(ASK THE FOLLOWING QUESTIONS IF THE OPIOID PRESCRIPTION WAS **NOT** PICKED UP)

How has the MedSMA℞T game influenced the way you might use prescription opioid medications?

How has the FMSP influenced the way you might use prescription opioid medications?

How has the MedSMA℞T game influenced the way you might store prescription opioid medications?

How has the FMSP influenced the way you might store prescription opioid medications?

How has the MedSMA℞T game influenced the way you might dispose of prescription opioid medications?

How has the FMSP influenced the way you might dispose of prescription opioid medications?

Is there anything else you would like to add?
